# Trapeziometacarpal Joint Arthroplasty: Medium-Term Clinical Outcomes and Survival

**DOI:** 10.7759/cureus.72888

**Published:** 2024-11-02

**Authors:** Guilherme Correia, Elisabete Ribeiro, Rodrigo Correia, Melanie Ribau, Pedro Varanda, Juvenália Ribeiro, Luís Filipe Rodrigues

**Affiliations:** 1 Orthopaedics and Traumatology, Hospital de Braga, Braga, PRT; 2 Northern Rehabilitation Center, Centro Hospitalar Gaia Espinho, Gaia, PRT

**Keywords:** basal thumb arthritis, maïa® prosthesis, rhizarthrosis, trapeziometacarpal joint arthroplasty, trapeziometacarpal osteoarthritis

## Abstract

Trapeziometacarpal joint (TMC) arthroplasty (TMA) is a treatment option for failed symptomatic treatment of basal thumb arthritis. This study aimed to evaluate clinical and radiological outcomes and complications of Maïa™ prosthesis (Groupe Lépine, Genay, France) since its introduction in our institution with a minimum of three years follow-up. We performed a retrospective study of patients with TMA treated with Maïa prosthesis. Between 2015 and 2017, 22 patients (21 female and one male patient) were submitted to TMA with Maïa prosthesis, with a mean age of 60.1±6.6 (95%CI 57.1, 63.0) years old. According to the Eaton-Littler classification, four patients had rhizarthrosis grade II, and 18 had grade III. The average follow-up was 56.4±6.0 months (95%CI 53.8, 59.1). Mean TMC radial abduction was 53.0±15.9º (95%CI 43.1, 62.3), corresponding to 89% (95%CI 78, 100) of the contralateral side. The mean Kapandji score was 9.5±0.7 (95%CI 9.1, 9.8), corresponding to 98% (95%CI 94,100) of the opposite side. The mean key pinch was 4.7±1.4 kg (95%CI 3.9,5.4), and the mean grip strength was 8 kg (95%CI 4, 12) against 10 kg (95%CI 6, 14), which corresponded to 79% (95%CI 70, 88), and 81% (95%CI 66, 96) of the non-operated side force, respectively. The mean satisfaction score with the procedure was 8.8±2.3 (95%CI 7.6, 10.0) out of 10. The mean qDASH (Quick Disabilities of the Arm, Shoulder, and Hand) score was 25.8±30.0 (95%CI 10.0, 40.7). The total revision rate was 13.6% (two cases of dislocation and one for loosening of the trapezium cup). There were no infections. The survival rate of the implant was 86.4% (95%CI 78.3, 93.3) after a five-year follow-up. There is still limited information regarding the long-term results of TMA using the Maïa prosthesis. TMA is a technically demanding procedure with a significant learning curve. With this study, we report satisfactory medium-term results in terms of motion, strength, and patient satisfaction.

## Introduction

Hand arthritis most commonly affects the trapeziometacarpal joint (TMC), making it the second most impacted joint in the hand. Typically affects post-menopausal women [1], with a prevalence of around 33%, but only one-third of the patients present with pain, restricted mobility, and reduced strength [2].

Several surgical techniques are available when nonsurgical modalities have failed. The surgical procedure of trapeziectomy with tendon interposition or suspension arthroplasty continues to be extensively employed, primarily for its efficacy in alleviating pain [3]. However, the limitations of this procedure may include slow postoperative recovery and weakness of the pinch strength compared with TMC arthroplasty (TMA) [4]. Compared to other surgical treatments, TMA, introduced in 1973 [5], had better strength, mobility, and a faster recovery time, with a preserved thumb length [6]. Since then, several improvements have been made in the design, cementless properties, and prosthesis modularity. TMA survival rates have steadily approached and aligned with those observed in total hip arthroplasty [7-9].

Due to the complications, costs, and lack of clear evidence showing that trapeziometacarpal prostheses outperform other surgical options, many experts still advocate trapeziectomy with ligament reconstruction and tendon interposition (LRTI) as the preferred approach. Additional medium- and long-term studies are necessary to evaluate and compare the outcomes of these treatments [6]. Nevertheless, the primary worries revolve around the risks of dislocation and the extended-term osteointegration of the cup. The purpose of this retrospective study was to assess medium-term outcomes of the Maïa™ prosthesis, focusing on complication, revision, and survival rates, and to compare these findings with existing literature.

## Materials and methods

This was a retrospective analysis conducted at Hospital de Braga, Braga, Portugal, of patients with TMA treated with the Maïa prosthesis. The study was approved by the Ethical Committee of Hospital de Braga (approval number: 40_2022).

We included patients above 18 years old at the surgery, primary TMC osteoarthritis, minimum follow-up of 36 months, stages II and III based on the Eaton classification, absence of prior TMC surgeries, and failure of the nonsurgical treatment. We excluded patients with radiographic evidence of scaphotrapeziotrapezoidal arthritis. Patients were followed up during the first year at three weeks, one month, three months, six months, and 12 months, and then annually thereafter. The clinical data presented pertains to the most recent follow-up. Patients who had undergone revision at the time of data collection were excluded from the clinical analysis, as the objective of the study was to assess the function of the implants that were still in place.

**Figure 1:**
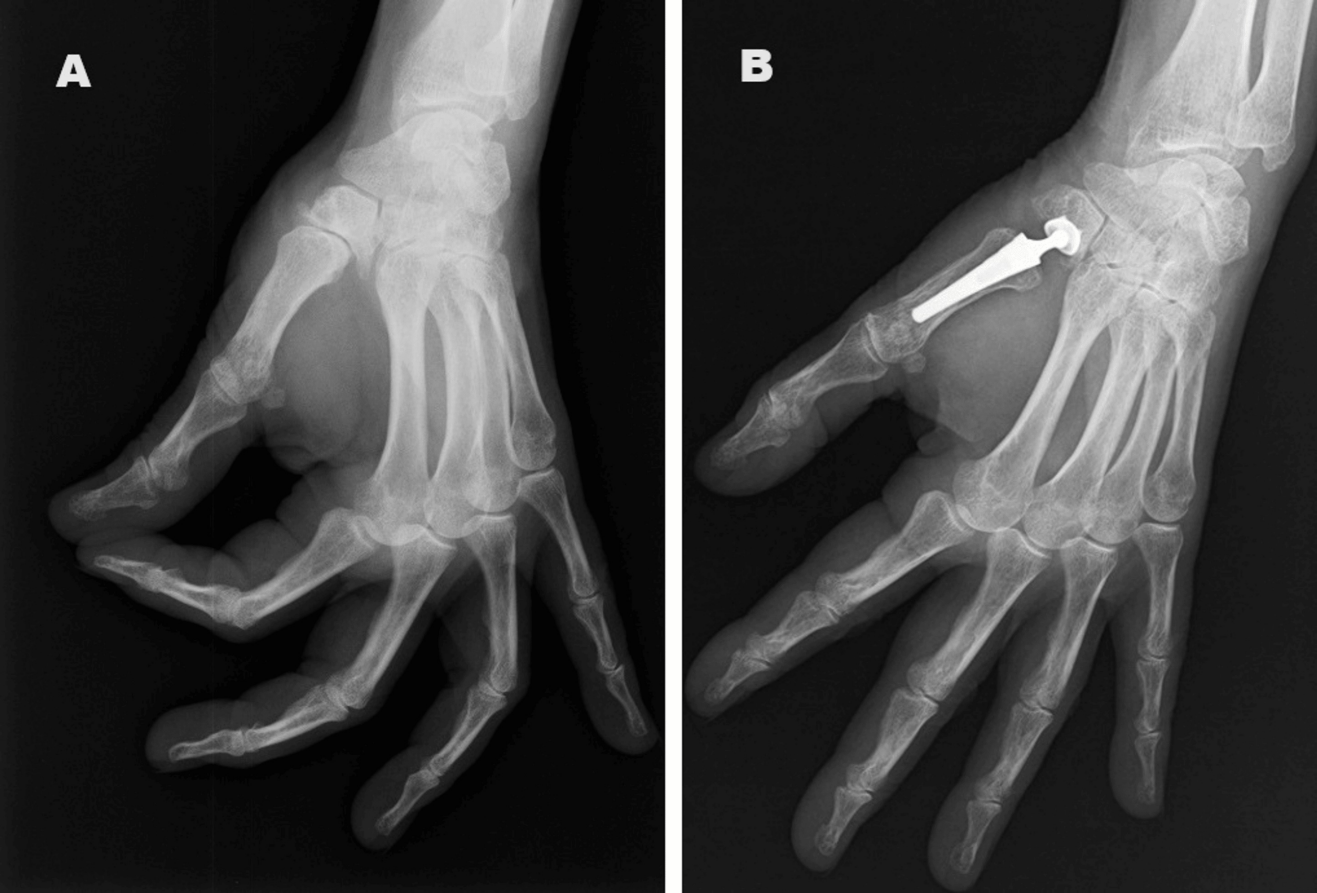
Preoperative (A) and postoperative (B) radiographs of the left hand in a patient with Eaton-Littler grade III rizharthosis submitted to a carpometacarpal arthroplasty with a Maïa™ implant (Groupe Lépine, Genay, France)

Surgeries were performed by two hand surgeons using the same surgical technique. The procedure started with a dorsolateral approach spanning approximately 3 cm, originating at the base of the thumb's dorsum and extending longitudinally towards the anatomical snuff box. The superficial radial nerve and its branches were carefully identified and retracted. Dissection continued between the extensor pollicis brevis and the abductor pollicis longus, taking care to avoid injury to the radial artery and its tributaries. After exposing the base of the metacarpal and the trapezium through a longitudinal capsulotomy, the metacarpal was first addressed, starting with a 5 mm cut on its base and another oblique cut from dorsal to volar at its volar base using an oscillating saw. The canal was prepared with the starter and progressive rasps, and the most suitable size was selected. The trapezium surface was then flattened by removing peripheral osteophytes. The entry point was found using a 2 mm Kirschner wire (K-wire) with fluoroscopy support. The trapezium was prepared with conic and spheric rasps, and the most suitable size was chosen. The definitive cup was inserted manually and according to press-fit. Only retaining trapezial cups (semi-constrained) were used. After definitive stem insertion, the most adequate neck size was chosen to achieve the greatest circumferential motion and thumb stability. At the end of the procedure, final radiographs were taken, followed by plentiful wound irrigation and anatomic closure (Figure 1).

To allow capsular healing and implant osteointegration, a splint was applied for three weeks. Immediate range of motion and physical rehabilitation started right afterward.

The range of motion was measured using a goniometer for the first web space (thumb abduction) and thumb opposition using the Kapandji Score. Bilateral grip and pinch strength were measured using a grip and pinch gauge (Sammons Preston Jamar Hydraulic Hand Finger Dynamometer). Preoperative measurements weren’t available. We asked the patients to complete the Portuguese version of the qDASH (Quick Disabilities of the Arm, Shoulder, and Hand) questionnaire [10]. A 10-point scale ranging from 0 (no satisfaction) to 10 (complete satisfaction) was used to measure patient satisfaction with both the surgical outcome and the appearance of the digit post-surgery. TMC arthritis stage, based on the Eaton-Littler classification, was assessed on preoperative radiographs. Postoperative radiographic variables included periprosthetic ossification, fracture, and loosening. Any complications were recorded from the patient’s medical record.

Descriptive statistics was used to summarize participant characteristics at baseline. Mean and standard deviation (SD) were calculated for continuous variables with normal distribution and reported frequencies and percentages for categorical variables. The parametric test used was the Independent-sample T-test (t). Categorical variables were compared through Chi-square tests (χ²) and post-hoc tests, such as Bonferroni correction, were employed to examine within-group and between-group differences. The Kaplan-Meier method was used to estimate implant survival. Statistical analyses were done using IBM SPSS Statistics for Windows, Version 25.0 (Released 2017; IBM Corp., Armonk, New York, United States). The level of significance was set at p < 0.05.

## Results

Between 2015 and 2017, 22 patients (21 female patients and one male) were submitted to Maïa TMA, with a mean age of 60.1±6.6 years (95%CI 57.1, 63.0). The mean follow-up time was 56.4±6.0 months (95%CI 53.8, 59.1). Six other procedures were performed simultaneously: five carpal tunnel releases and one distal interphalangeal arthrodesis. The mobility and strength results were compared to the contralateral side in patients with a unilateral prosthesis. Clinical results are detailed in Tables 1, 2.

**Table 1:** Clinical results in detail. N.A.: not applicable; VAS: visual analog scale; qDASH: Quick Disabilities of the Arm, Shoulder, and Hand

Patients	Operated side	VAS (0-10)	qDASH (0-100)	Global satisfaction score (0-10)	Cosmetic appearance satisfaction score (0-10)	Revision (yes/No)
Patient 1	Right	3	25.0	9	9	No
Patient 2	Left	1	0.0	10	10	No
Patient 3	Left	1	0.0	9	10	No
Patient 4	Left	1	15.9	10	10	No
Patient 5	Left	1	0.0	10	10	No
Patient 6	Left	N.A.	N.A.	N.A.	N.A.	Yes
Patient 7	Bilateral	1	45.5	8	10	No
Patient 8	Left	1	0.0	10	10	No
Patient 9	Right	4	79.5	10	10	No
Patient 10	Left	N.A.	N.A.	N.A.	N.A.	Yes
Patient 11	Bilateral	1	0.0	10	10	No
Patient 12	Left	4	65.9	10	10	No
Patient 13	Left	3	29.5	10	10	No
Patient 14	Right	N.A.	N.A.	N.A.	N.A.	Yes
Patient 15	Right	1	15.9	10	10	No
Patient 16	Left	1	5.0	10	10	No
Patient 17	Right	N.A.	N.A.	N.A.	N.A.	No
Patient 18	Right	1	0.0	10	10	No
Patient 19	Bilateral	4	55.0	6	10	No
Patient 20	Left	1	0.0	10	10	No
Patient 21	Left	5	90	2	10	No
Patient 22	Right	1	37.5	N.A.	N.A.	No
Mean	N.A.	2.1	25.8	8.8	9.7	N.A.
Standard deviation	N.A.	1.6	29.9	2.3	1.2	N.A.

**Table 2:** Clinical results in detail (continued). N.A.: not applicable; Op: operated; Ct: contralateral

Patients	Grip strength (Kg)			Pinch strength (Kg)			Radial abduction (degrees)			Opposition (Kapandji)		
	Op side	Ct side	% Op side	Op side	Ct side	% Op side	Op side	Ct side	% Op side	Op side	Ct side	% Op side
Patient 1	13.3	17.3	77%	5.0	6.0	83%	35	55	64%	9	10	90%
Patient 2	N.A.	N.A.	N.A.	N.A.	N.A.	N.A.	N.A.	N.A.	N.A.	N.A.	N.A.	N.A.
Patient 3	4.0	4.7	86%	3.8	5.8	66%	45	40	113%	9	9	100%
Patient 4	0.7	0.7	100%	3.2	4.2	76%	40	65	62%	10	10	100%
Patient 5	21.3	26.0	82%	7.3	7.2	102%	40	45	89%	10	10	100%
Patient 6	N.A.	N.A.	N.A.	N.A.	N.A.	N.A.	N.A.	N.A.	N.A.	N.A.	N.A.	N.A
Patient 7	18.7	N.A.	N.A.	6.7	N.A.	N.A.	65	N.A.	N.A.	10	N.A.	N.A
Patient 8	9.3	10.0	93%	4.8	5.3	91%	55	70	79%	10	10	100%
Patient 9	7.0	5,3	131%	4.5	5.5	82%	55	55	100%		10	100%
Patient 10	N.A.	N.A.	N.A.	N.A.	N.A.	N.A.	N.A.	N.A.	N.A.	N.A.	N.A	N.A
Patient 11	16.7	N.A.	N.A.	6.2	N.A.	N.A.	35	N.A.	N.A.	10	N.A	N.A
Patient 12	N.A.	N.A.	N.A.	N.A.	N.A.	N.A.	N.A.	N.A.	N.A.	N.A.	N.A	N.A
Patient 13	9.3	10.7	88%	4.8	4.8	100%	75	75	100%	10	10	100%
Patient 14	N.A.	N.A.	N.A.	N.A.	N.A.	N.A.	N.A.	N.A.	N.A.	N.A.	N.A	N.A
Patient 15	1.3	6.0	22%	2.7	5.0	53%	45	60	75%	9	10	90%
Patient 16	1.7	2.0	83%	2.5	4.5	56%	45	60	75%	8	8	100%
Patient 17	N.A.	N.A.	N.A.	N.A.	N.A.	N.A.	N.A.	N.A.	N.A.	N.A.	N.A	N.A
Patient 18	16.0	18.0	89%	6.2	7.7	80%	90	85	106%	9	9	100%
Patient 19	6.5	N.A.	N.A.	3.5	N.A.	N.A.	55	N.A.	N.A.	10	N.A	N.A
Patient 20	5.3	6.7	80%	5.2	6.3	82%	65	55	118%	10	9	111%
Patient 21	5.7	11.3	50%	4.8	6.2	78%	55	60	92%	9	10	90%
Patient 22	7.7	10.7	72%	3.8	5.0	77%	40	45	89%	9	10	90%
Mean	9.0	10.0	81.0%	4.7	5.7	78.9%	52.5	59.2	89.2%	9.5	9.6	97.8%
Standard deviation	6.4	7.1	25.3	1.4	1.0	14.5%	15.3	12.6	17.8%	0.6	0.7	6.1%

Mean TMC radial abduction was 53.0±15.9º (95%CI 43.1, 62.3), corresponding to 89% (95%CI 78, 100) of the contralateral side. The mean Kapandji score was 9.5±0.7 (95%CI 9.1, 9.8), corresponding to 98% (95%CI 94, 100) of the opposite side. The mean key pinch was 4.7±1.4 kg (95%CI 3.9, 5.4) against 5.7±1.0 kg (95%CI 5.0, 6.3) for the non-operated side (t(15)=13.3, p <0.001), and mean grip strength was 8 kg (95%CI 4, 12) against 10 kg (95%CI 6, 14) for the non-operated side (t(15)=5.6, p <0.001), which corresponded to 79% (95%CI 70, 88) and 81% (95%CI 66, 96) of the non-operated side force, respectively. The mean satisfaction score with the procedure was 8.8±2.3 (95%CI 7.6, 10.0) out of 10, and 15 patients were very or completely satisfied with the outcome. The mean qDASH score was 25.8±30.0 (95%CI 10.0, 40.7).

**Figure 2:**
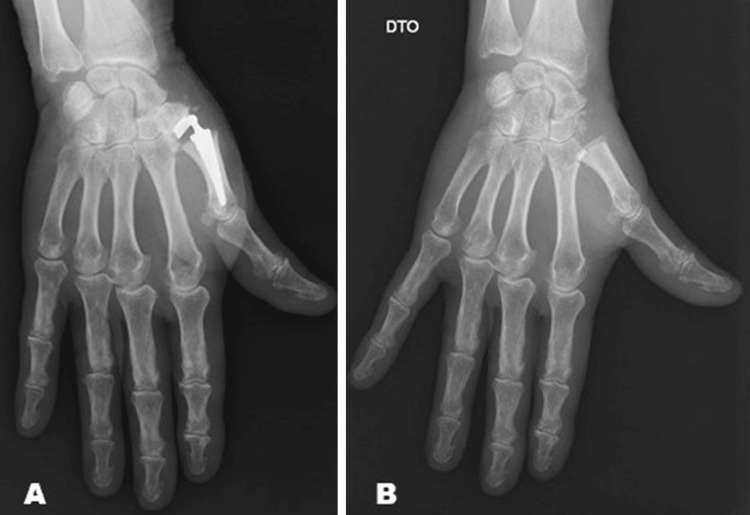
Patient with implant instability that required revision. A: Carpometacarpal arthroplasty of the right hand with implant instability; B: Revision surgery that consisted on implant removal, trapeziectomy, and suspension ligamentoplasty.

**Figure 3:**
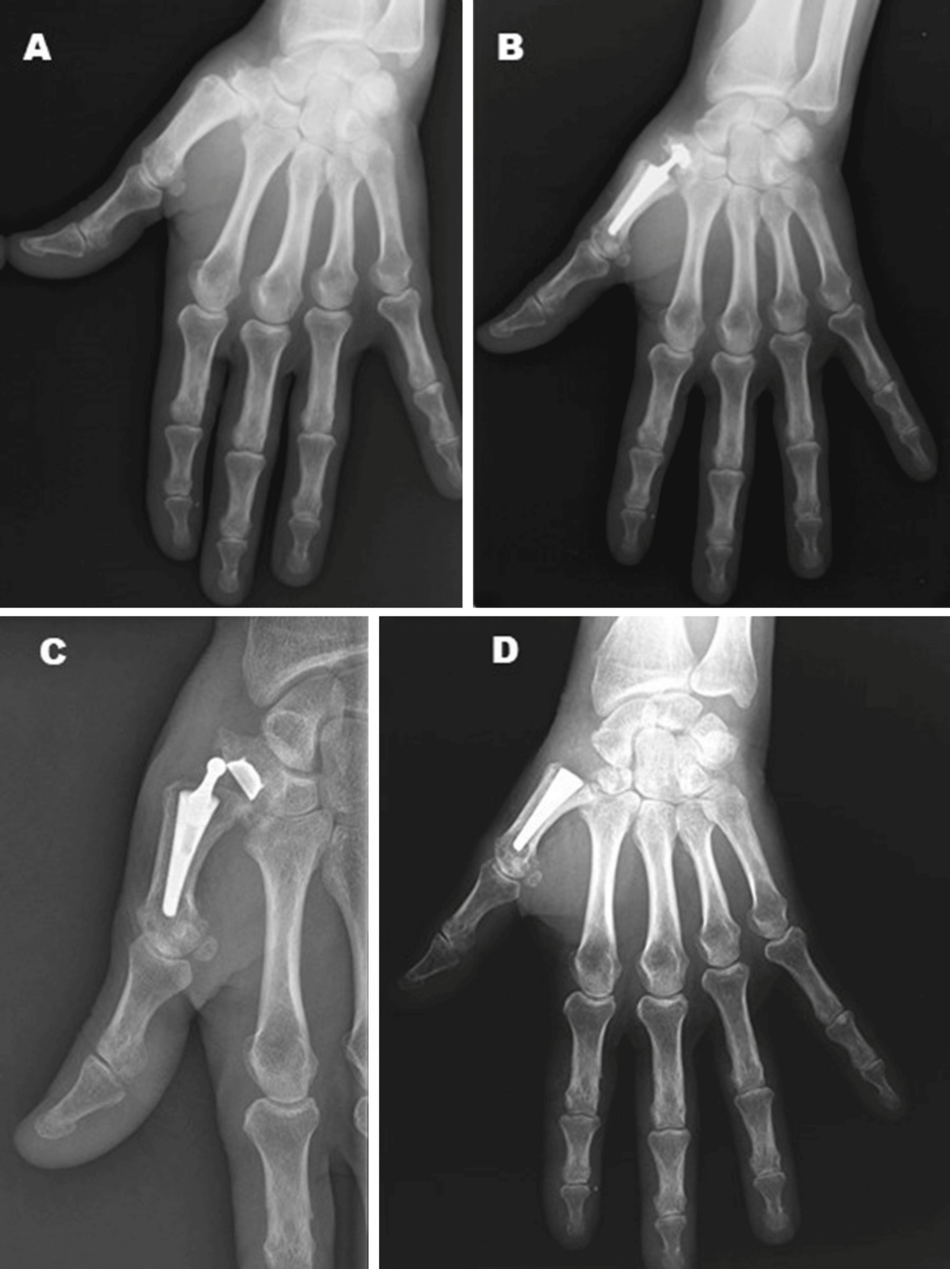
Patient with implant instability that required revision with implant stem retention. A: Rhizarthrosis Eaton Littler grade III on the left hand; B: Carpometacarpal arthroplasty with Maïa implant; C: Implant dislocation; D: Revison surgery with impant stem retention, trapeziectomy and suspension ligamentoplasty.

Dislocation was the most frequent complication in our series, occurring in two cases (9.1%). Also, one patient (4.5%) had a loosening of the trapezial cup. These three patients (13.6%) were submitted to revision, which consisted of extraction of the implant components and trapeziectomy (Figure 2) except for the metacarpal stem when well-integrated since its removal would trigger more significant morbidity (Figure 3). Abductor Pollicis Longus “Hammock” ligamentoplasty was then performed according to the description of Mathoulin [11]. Implants that had not been revised were considered to be survivors. The mean survival time was 66.8 months (95%CI 63.5, 70.0). After a five-year follow-up, 19 out of 22 patients still had a functioning implant (survival rate of 86.4% (95%CI 78.3, 93.3). No periprosthetic ossifications or other complications were reported.

A comprehensive analysis of patient factors between the revised and non-revised populations was conducted, revealing no significant risk factors associated with age (t(20)=0.01, p=0.766, 95%CI -10.00, 7.48), operated side (χ²(1, N = 22) = 0.014, p=0.907), sex (χ²(1, N = 22) = 0.165, p=0.684), or Eaton-Littler classification (χ²(1, N = 22) = 0.737, p=0.380).

## Discussion

Limited information exists regarding the long-term results of TMC prostheses. TMA is a technically demanding procedure with a significant learning curve. Correct patient selection and surgical expertise are essential for achieving the best outcomes and minimizing complications. We aimed to report our institution's medium-term clinical results after Maïa TMA.

Clinical results regarding motion, satisfaction, and qDASH score at the final follow-up were generally good. Both motion and strength parameters are around the 80% barrier of the contralateral side. Although both pinch and grip strength were slightly lower than the non-operated side (4.7±1.4 kg vs. 5.7±1.0 kg with p<0.001 for pinch strength and 9.0±6.4 kg vs. 10.0±7.1 kg with p<0.001 for grip strength), they both remained quite acceptable relative to average values [12]. Additionally, the Kapandji score, which gives a notion of thumb mobility, was very similar to the non-operated hand (98%). These results translate into an excellent overall function. Patients’ perception of their function based on qDASH of 25.8±30.0 state the final good results.

According to a systematic review by Holme et al. in 2021 [13], the Maïa prosthesis was reported in five studies [4,14-17], all of which were descriptive cohorts, with a total of 451 replacements. In a study utilizing cumulative survival analysis, the reported survival rates indicated a 93% survival at the five-year mark [4]. At a mean of 59 months follow-up, failure rates were 9.8%, loosening rates 2.4%, and dislocation rates 4.4% [13].

When implanting TMAs, the most significant complications frequently contribute to revision, such as loosening or implant dislocation, the latter the most common cause of early revisions [18]. In our study, the main complication encountered was prosthesis dislocation, which occurred in two patients (9.1 %). This rate was higher than in previous studies (Table 3) except in a study by Andrzejewski et al. [14]. Generally, dislocations can be caused by trauma, but many dislocations occur with no apparent reason [15]. In our cohort, despite the fact of implanting only semi-constrained cups, two patients suffered dislocation without known trauma. Semi-constrained cups are intended to provide stability that can help prevent early dislocation during the initial period of tissue healing. However, according to Caekebeke et al., the use of constrained cups does not seem to protect against dislocation; according to the authors, the adequate placement centrally in the bone and within the center of joint mobility appears to play the most crucial factor for prosthesis stability [15]. Duerinckx et al. proposed that the prosthetic cup in the trapezium should be positioned parallel to the proximal articular surface of the trapezium [19]. Additionally, they recommend combining it with a metacarpal stem featuring a 7° palmar offset. This allows full physiological thumb motion within the constraints of current cup designs and should minimize the risk of stem dislocation and reduce eccentric wear. Correct cup alignment can be checked intra-operatively with fluoroscopy, which was systematically done in our cohort.

**Table 3:** Results from cohorts using the same implant for trapeziometacarpal arthroplasty. qDASH: Quick Disabilities of the Arm, Shoulder, and Hand; DASH: Disabilities of the Arm, Shoulder, and Hand; N.A.: not available; Post=op; postoperative; NOp: non-operated; Op: operated

Cohort (year)	N	Mean Age	Months of follow-up, mean (range)	Post-op qDASH/ DASH	Abduction	Kapandji	Pinch (Kg)	Pinch ratio Op/NOp	Dislocation	Loosening	Revision	Survival rate
Current study	22	60	56.4 (46-70)	qDASH 25.8	53°	9.4	Op 4.6 NOp 5.7	81%	9.1%	4.5% (cup)	13.6%	86.4% at 5 years
Andrzejewski and Ledoux (2019) [14]	113	59.5	63 (32-143)	DASH 26.7	44°	8.9	Op 4.8 NOp 5.4	89%	10%	2%	12%	92% at 5 years
Caekebeke and Duerinckx (2018) [15]	50	57	65 (56-71)	DASH 7	N.A.	9	Op 7 NOp 7	100%	0%	0%	4%	96% at 65 months
Toffoli and Teissier (2017) [4]	96	68	76 (60-102)	qDASH 17.5	33°	9.2	5.6	N.A.	1%	4% (cup)	8%	93% at 5 years
Bricout and Rezzouk (2016) [16]	156	62.7	37.8 (13.4-71.0)	qDASH 14.3	N.A.	N.A.	N.A.	N.A.	4%	3% (cup)	12%	90.8% at 62 months
Kubát and Trtík (2012) [17]	36	60	42 (37-?)	qDASH 22.5	N.A.	N.A.	N.A.	N.A.	3%	3% (cup)	6%	N.A.

We reported one case of aseptic loosening (4.5%) (Table 3). Toffoli et al. reported a 4.2% rate, with all cases occurring in the first 40 months, suggesting that osteointegration failed [4]. Excessive loads exerted on the implant and transmitted to the cup-bone interface could be the primary cause, hindering osteointegration. We believe it could be enhanced by the semi-retaining character of the cup used in our study. The poor quality of the osteoporotic trapezium bone may be a biological factor leading to trapezium failure [4]. Bricout et al. recommended that any doubt regarding the trapezial bone quality during the procedure should pose a good enough reason for TMA to be contraindicated [16].

The survival of the TMA is a primary concern for hand surgeons. The uncertainty about survival can prompt surgeons to choose other surgical options for treating basal thumb arthritis. The possibility of surgical revision must be discussed during the preoperative patient information phase. If the TMC prosthesis fails, trapeziectomy is still an option, while the reverse is not possible. Consequently, initially performing TMA provides more options in the future than doing a primary trapeziectomy [16]. Additionally, in case of failure, the outcome of secondary trapeziectomy is comparable with that of primary trapeziectomy [20]. In the present study, the five-year survival rate was 86.4%, with three patients submitted to revision. This result is slightly lower than the previously reported in the literature at five years of follow-up (Table 3). All the patients were submitted to trapeziectomty and ligament reconstruction using abductor pollicis longus with the recovery of their complaints.

Research has shown that not all complications can be attributed to flawed implant designs or patient factors. It is widely acknowledged that the incidence of surgical errors tends to diminish as a surgeon gains experience, a principle that is well-established in the field of arthroplasty [21]. One study noted that complication rates were significantly elevated during the initial 30 procedures due to technical mistakes, but these rates dropped considerably in subsequent cases [22]. This pattern is consistent with observations made in hip, knee, and shoulder arthroplasties [23-25]. In this report, we present the first 22 cases of TMA using this implant after its introduction in our department, which may in part explain a higher dislocation rate. Some studies now report TMA 10-year revision rates, but they do not yet include newer implant designs like the Maïa [26], which currently has follow-up data primarily up to five years (Table 3). Therefore, continued analysis in the coming years is essential to gain a better understanding of the long-term outcomes of this treatment option with this implant design.

The present study has some considerable limitations. First, as a retrospective study, preoperative clinical data are lacking. This is a significant drawback, as the isolated time frame precludes the analysis of the potential changes with the procedure. Another limitation was the low number of patients, which may partly explain the inflated failure rates. The other limitation was that not all patients could be clinically reviewed for mobility and strength tests.

## Conclusions

This study provides valuable medium-term clinical insights into the outcomes of Maïa TMA, demonstrating good overall function and patient satisfaction. While the procedure appears to yield acceptable strength and motion outcomes, the observed complication rates, notably dislocation, underscore the importance of surgical experience and precise implant positioning. Future research should focus on the long-term durability of newer implant designs like Maïa TMA, as well as techniques that may mitigate common complications, such as loosening and dislocation. Moreover, as emerging technologies and refined surgical techniques continue to evolve, studies comparing these options can potentially enhance the procedure's efficacy and safety profile over time. A patient-centered approach remains paramount in selecting treatment options, especially considering factors such as age, bone quality, and comorbidities. Tailoring surgical decisions to align with individual patient needs, functional goals, and lifestyle expectations is critical to optimizing outcomes and satisfaction, particularly in elderly patients or those with significant health considerations. Expanding the evidence base on TMA with diverse patient populations and longer follow-up periods will support more informed, individualized surgical decisions for managing thumb carpometacarpal arthritis effectively.
